# CreelCat, a Catalog of United States Inland Creel and Angler Survey Data

**DOI:** 10.1038/s41597-023-02523-2

**Published:** 2023-11-03

**Authors:** Nicholas A. Sievert, Abigail J. Lynch, Holly S. Embke, Ashley Robertson, Mitchel Lang, Anna L. Kaz, Matthew D. Robertson, Stephen R. Midway, Lyndsie Wszola, Craig P. Paukert

**Affiliations:** 1 U.S. Geological Survey, Oak Ridge Institute for Science and Education, Reston, VA 20192 USA; 2 U.S. Geological Survey, National Climate Adaptation Science Center, Reston, VA 20192 USA; 3 U.S. Geological Survey, Midwest Climate Adaptation Science Center, Saint Paul, MN 55108 USA; 4 George Mason University, Department of Environmental Science and Policy, Fairfax, VA 22030 USA; 5 University of Virginia, Department of Environmental Sciences, Charlottesville, VA 22904 USA; 6 Department of Oceanography and Coastal Sciences, Louisiana State University, Baton Rouge, LA 70803 USA; 7 Centre for Fisheries Ecosystems Research, Fisheries and Marine Institute of Memorial University of Newfoundland, P.O. Box 4920, St. John’s, NL A1C 5R3 Canada; 8 Missouri Cooperative Fish and Wildlife Research Unit, The School of Natural Resources, University of Missouri, Columbia, Missouri 65211 USA; 9 U.S. Geological Survey, Missouri Cooperative Fish and Wildlife Research Unit, The School of Natural Resources, University of Missouri, Columbia, Missouri 65211 USA

**Keywords:** Databases, Freshwater ecology, Environmental social sciences, Ecosystem services

## Abstract

The United States Inland Creel and Angler Survey Catalog (CreelCat) contains a national compilation of angler and creel survey data collected by natural resource management agencies across the United States (including Washington, D.C. and Puerto Rico). These surveys are used to help inform the management of recreational fisheries, by collecting information about anglers including what they are catching and harvesting, the amount of effort they expend, their angling preferences, and demographic information. As of May 1, 2023, CreelCat houses over 14,729 surveys from 33 states, Puerto Rico, and Washington, D.C., comprising 235 data fields across 8 tables. These tables contain 235,015 records of fish catch and harvest metrics, 27,250 angler preference metrics, 14,729 records of survey characteristics, 13,576 records of effort metrics, and 409 records of angler demographics. Though individual creel surveys are often deployed to meet local science and management objectives, creel data aggregated across jurisdictions has the potential to address larger scale research and management needs.

## Background & Summary

Angler surveys are used by natural resource management agencies to collect data related to recreational angling. They are typically conducted for a waterbody over a period of time ranging from several months to a year and are used to collect information on the types and numbers of fish that are captured and harvested, how much time anglers spend fishing, as well as the preferences and demographics of those anglers^1–3^. A variety of methods are used to conduct angler surveys, otherwise referred to as creel surveys, including those which contact anglers via mail, phone, or the internet; programs which encourage anglers to report their activities, such as diaries or dropbox cards; or angler-intercept surveys. Angler-intercept surveys are conducted via on-site interviews to gather information while also conducting angler counts which are used to generate overall estimates of a variety of metrics^4^. Angler surveys are often used to inform management decisions such as length and harvest limits, stocking practices, infrastructure development, and economic impact assessments^5–7^. In addition to having enormous utility for these local efforts, when angler surveys are compiled across broad spatial and temporal scales they also have the potential to inform large-scale research and management questions^8^. The objective of our effort was to compile these data for inland recreational fisheries across the United States to help support the needs of managers and researchers.

While there is still often a need for management and decision making to occur at the local level, there is an increasing recognition that many processes taking place across large spatial scales impact fish and anglers. In many systems, anglers can make decisions about where to fish among waterbodies over a relatively large spatial extent^9^. Factors including fish population characteristics, fish community composition, travel distances, accessibility, infrastructure, as well as waterbody and landscape characteristics, all shape how anglers allocate effort across the landscape^10^. To effectively manage fish populations and provide opportunities for recreational angling, management agencies are placing an emphasis on managing fisheries as large-scale social-ecological systems^4,11,12^. Management of waterbodies across large spatial scales can enhance the diversity of opportunities for anglers with varied preferences, but large-scale datasets that span the spatial and temporal extents being evaluated are needed to support these efforts.

Climate change is exerting an influence on recreational fisheries in the United States and across the world. In some systems, increasing water temperatures are altering habitat suitability for popular game species like Salmonids, *Sander vitreus* (Walleye), and *Micropterus dolomieu* (Smallmouth Bass)^13^. Changes in temperature may also influence factors such as prey availability, growth rates, and timing of spawning, all of which may influence how fisheries are managed^14–17^. Shifts in fish community composition or population characteristics may influence the behavior of recreational anglers. In addition to impacting the fish themselves, a changing climate may influence angler behavior directly. Changes in conditions associated with climate (e.g., decreasing ice duration, heat waves) may lead to shifts in when or if anglers choose to participate^15,18,19^. Large-scale datasets can help researchers better quantify associations between broad-scale patterns in climate and fishery characteristics to better understand the impacts of climate change on recreational angling.

The impact of shifting angler demographics on fisheries resources is also a key knowledge gap. Angler recruitment, retention, and reactivation (R3) activities are prioritized by management agencies around the United States and require an understanding of the motivations to participate in angling^20^. Consideration of the impacts of population shifts into more urban and suburban environments is gaining traction as a research topic in the management of recreational fisheries^21^. In some areas, aging populations of anglers have been linked to changes in the use of fisheries^22^. Shifts in species preferences of anglers, angler motivations (e.g., harvest vs. catch and release; trophy vs. opportunity), and rates of harvest and release, all have the potential to reshape how fisheries are managed^23–29^. A large-scale compilation of angler survey data can be used to evaluate how factors such as angler preferences and demographics impact the angling opportunities that resource users desire and how changes in demographics may influence management decisions.

**Fig. 1:**
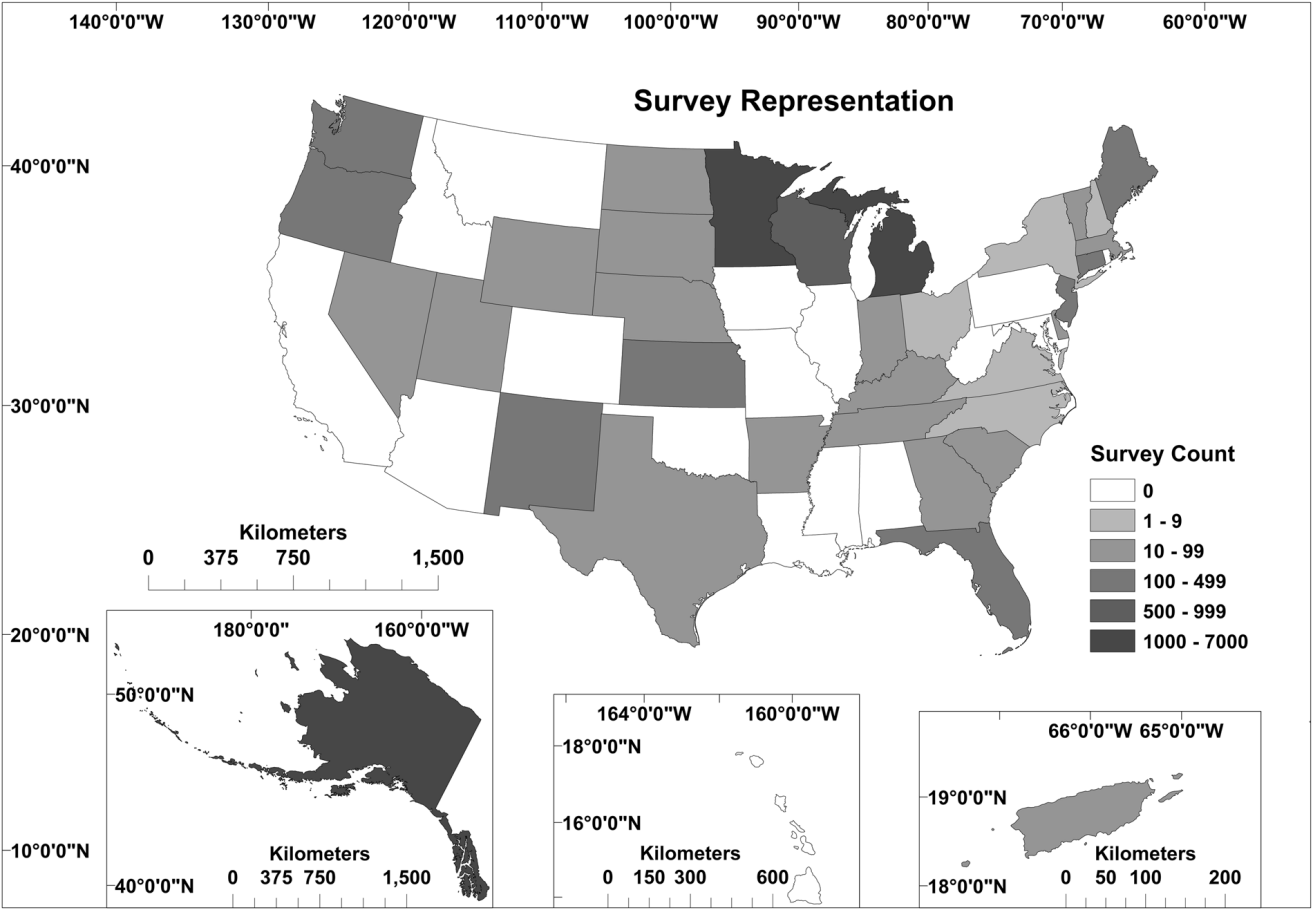
Map of number of surveys (waterbody × discrete time period) by state, territory, and District of Columbia in the United States Inland Creel and Angler Survey Catalog (CreelCat version 1.0: 10.5066/P9DSOPHD).

**Fig. 2:**
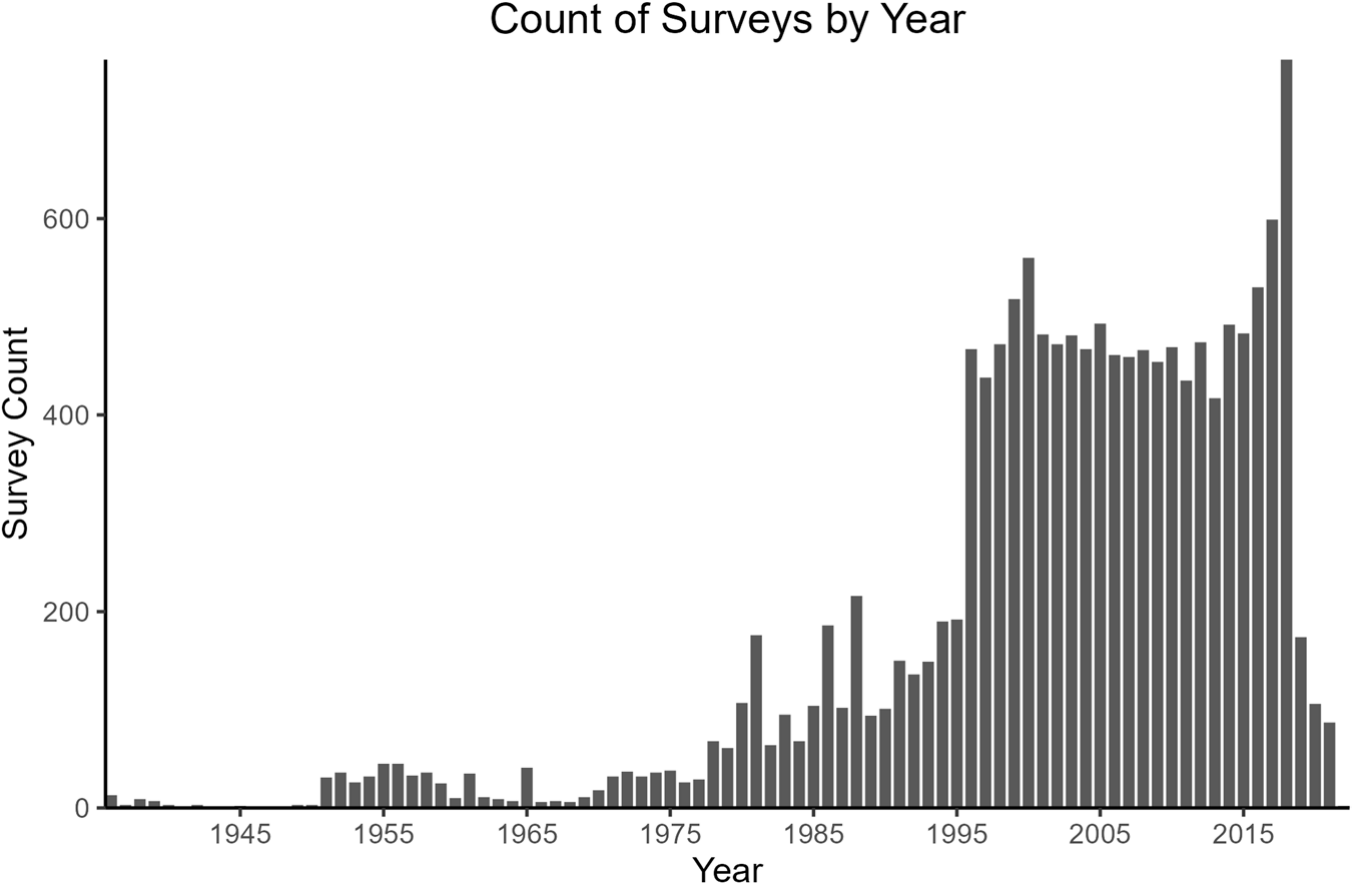
Count of surveys contained in the United States Inland Creel and Angler Survey Catalog (CreelCat version 1.0: 10.5066/P9DSOPHD) by year.

Here, we describe the United States Inland Creel and Angler Survey Catalog (CreelCat)—a repository of compiled angler survey data which may be used to address pressing management and research questions across broad spatial and temporal scales^30^. The data contained in CreelCat, as of May 1, 2023, provide access to angler survey results from 33 states, Washington, D.C., and Puerto Rico (Fig. 1). Data requests targeted data from 2010 to present but, where possible, historical records were included to provide additional long-term context (Fig. 2). Due to variation between survey methodologies among and within states, not all fields were populated for each survey. This variability included differences in which fields were recorded, the timing and duration of surveys, data collection and estimation methods, levels of taxonomic attribution, and units of measurement (which were standardized within CreelCat). Across the entire dataset there were records for 14,729 surveys with 235 characterized fields across 8 tables. Information characterizing fish catch and harvest contains 235,015 records (survey × taxa), 27,250 records (survey × taxa) of angler preference metrics, 14,729 records (survey) of survey characteristics, 13,576 records (survey) of effort metrics, and 409 records (survey) of angler demographics. To provide spatial context and link to additional data sources, shapefiles containing the georeferenced locations (points) and extents of surveys (polygons for lakes, reservoirs, ponds, and tailraces; lines for rivers and streams) are also included with the dataset.

## Methods

We developed CreelCat using a five-step process: develop objectives and a database schema, compile data, input data, attribute spatial information, and carry out calculations.

### Development of objectives and a database schema

We held a virtual workshop on May 21, 2020, to help identify the need for and potential use of these data for inland fisheries management and research. The workshop included participants from state natural resource management agencies (n = 7), federal agencies (n = 3), academia (n = 9), and multiple affiliations (n = 4). We had relatively broad geographic representation with participants from 15 states. Discussions regarding which survey types and information to include, how to structure the dataset, value for managers and researchers, and planning for longevity informed our plans for the effort. In order to solicit feedback, participants shifted between discussion of predetermined questions, intended to help steer the effort, in small breakout groups (4–6 participants) and forming a consensus among all participants in the full group. Based on these discussions we drafted a white-paper which was used to guide our efforts in developing CreelCat.

**Fig. 3:**
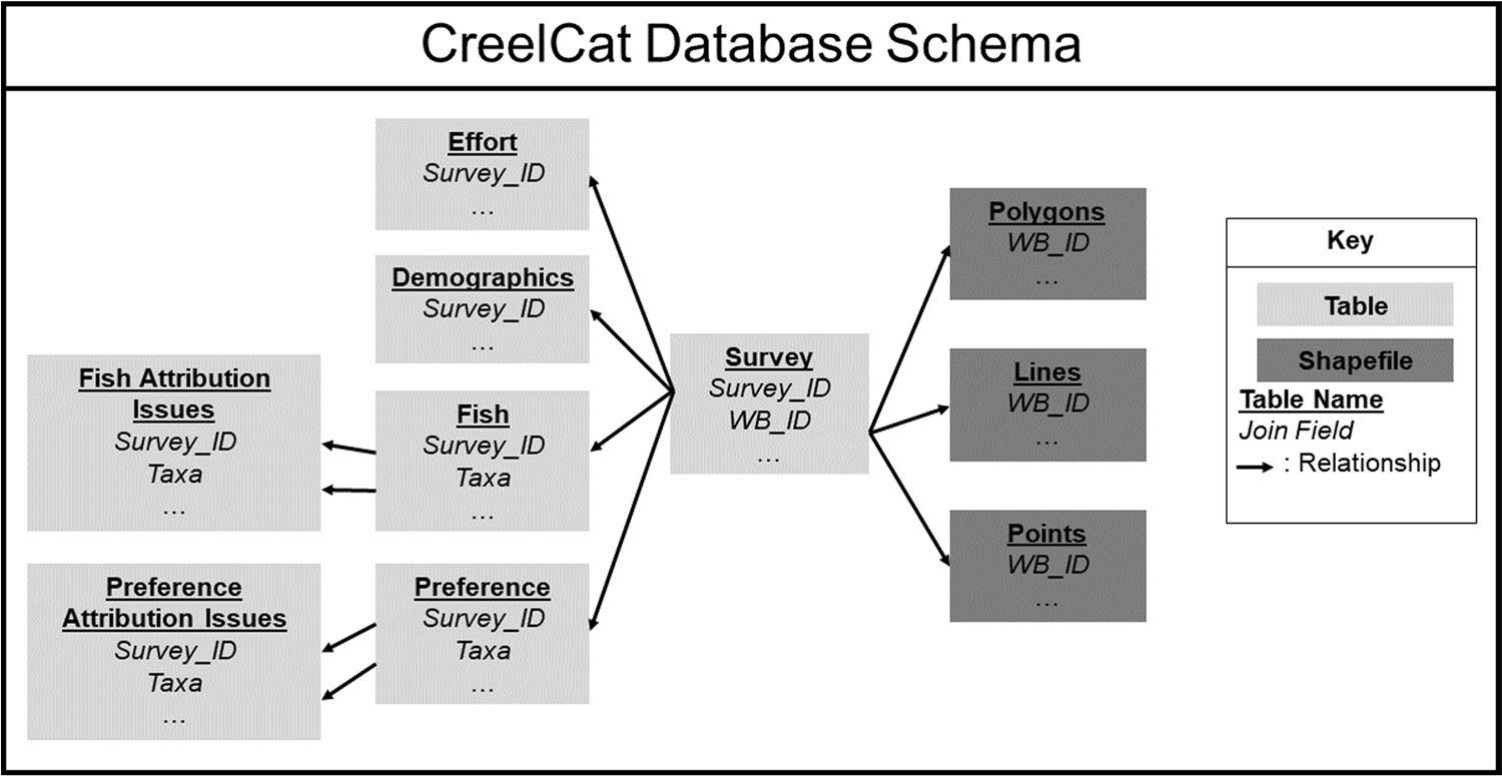
Relationships among United States Inland Creel and Angler Survey Catalog (CreelCat) tables and geospatial data. See Supplementary Table 1 for definitions of fields.

Based on feedback from the workshop, we prioritized the inclusion of angler-intercept surveys whenever possible but accepted other survey types when those were not available to ensure maximum spatial and temporal representation. We prioritized surveys which generated estimates over surveys that were limited to reporting raw interview-only data, and surveys that were linked to waterbodies over surveys tied to more general spatial areas (i.e., a state or region). The workshop group identified the following types of information relevant for inclusion in the database: survey and waterbody characteristics, angler effort, catch and harvest of fish, angler demographics, and angler preferences. We captured information for each of these areas in separate tables which we related to one another using an identifier unique to each survey (Fig. 3). Where possible, we attributed surveys geospatially to the dataset using an identifier unique to each survey area.

### Compiling creel and angler survey data

We contacted natural resource management agencies responsible for fisheries management in all fifty states as well as Washington, D.C. and Puerto Rico. Our inquiries for survey data outlined the types of data being requested and how they were to be used. All data included in the dataset received written permission from the data provider to be shared publicly. The format of data provided for inclusion in CreelCat included report and correspondence documents (i.e., pdf and Microsoft Word documents), electronic tabular data (i.e., CSV and Microsoft Excel files), and relational databases (i.e., Microsoft Access database). Copies of all data were preserved in their original form to ensure the integrity of the data as a reference source. Because the source data for this effort have been compiled from thousands of published reports as well as unpublished (but vetted and approved) internal datasets, it is impractical to reference them directly within this manuscript. However, within the dataset itself, the ‘Survey_Data’ table contains the field ‘Report_Citation’ which includes citations for the report associated with a given survey, the ‘Survey_Link’ field contains the URL associated with any datasets which can be accessed directly online, and the ‘Agency’ field contains the name of the source of the data.

### Data input

We reviewed all data provided to determine whether they were suitable for inclusion in the database or whether additional information was needed. This review included determining whether critical survey characteristics were documented such as the location, timing, and methodology, and whether sufficient documentation was provided to reliably interpret all fields (i.e., field definitions, units of measurement, ambiguous terminology). When any information was missing or unclear in the provided datasets, we asked the data providers for clarification or additional documentation. Any additional information provided was then stored with the source data and used to ensure all data were appropriately and accurately represented within CreelCat.

The methodology used for data entry was based on the format of the source data. All data were received as electronic files including report documents (.pdf and .doc files), tabular datasets (.csv and.xlsx), and relational databases (.accdb). We processed data contained in report documents by copying and pasting tables from the documents into a spreadsheet where the data could then be reformatted to match the format of CreelCat and transferred into the appropriate database tables. In cases where values/information were contained within text or tables which were not electronically readable (e.g., blurry photocopies, misaligned columns), data were manually transcribed from the source data reports into the appropriate database tables. We reformatted source data contained in electronic tabular formats within a spreadsheet and transferred to the appropriate database tables. We developed custom queries for any source datasets provided as relational databases to export data in a format which matched CreelCat and transferred it to the appropriate database tables.

We standardized data upon entry into CreelCat. This included converting taxonomic names to a single, shared nomenclature based on the US Geological Survey – Smithsonian Institution Integrated Taxonomic Information System (ITIS)^31^. The most widely used common names within our source data were selected for each taxa to accompany the scientific names (i.e., Burbot was selected as the common name for *Lota lota* over less frequently used names such as eelpout, lingcod, and coney-fish). For hybrid taxa, we assigned the common names used for both parental species. In some cases, surveys included data on non-taxonomic variants (i.e., Steelhead Rainbow Trout (*Oncorhynchus mykiss*) which are not taxonomically distinct but are often represented separately within fisheries datasets). Additionally, some metrics are attributed to groups which are not monophyletic such as “Trout” or “Panfish” which necessitated the inclusion of a groups category for taxonomic inputs. We standardized any measurement units to United States customary units which were the most commonly used units in the provided datasets.

### Spatial attribution

For records that included adequate documentation of where surveys took place, we georeferenced the location and, where adequate information was available, the survey extent. All spatial information can be linked to the tabular data via the waterbody identifier field, ‘WB_ID’, contained in the ‘Survey_Data’ table. A point shapefile documents representative locations for surveys. For surveys of lakes, reservoirs, ponds, and tailraces, we represented survey extents within a polygons shapefile, while we represented survey extents of rivers and streams within a lines shapefile.

Polygons and lines representing waterbody survey extents were either based on shapefiles provided by the data source, by linking surveys to waterbodies found within the National Hydrography Dataset (1:24k Version)^32^ waterbody or data source provided shapefiles, or by manually constructing polygons around survey extents or lines along stream path based on information provided in the source data and aerial imagery from the basemap in ArcGIS 10.2. This information represents the spatial extent of the surveyed waterbody based on the information provided in the source data. We calculated the area (polygons) and length (lines) to represent the spatial extent of the surveys.

We developed point-only data representing the locations of surveyed waterbodies to represent the locations of waterbodies which did not have the information needed to attribute a spatial extent represented by either a polygon or line. Point locations were typically based on either coordinates or location descriptions within the source data or from geospatial data provided by the source agency. Polygons and lines were converted to points in ArcGIS 10.2 and merged with the point only data to create the point layer which contains the location of all georeferenced surveys in CreelCat.

### Calculations

We calculated rate and percent-based metrics using R^33^. Examples include solving for either ‘Catch’, ‘Harvest’, or ‘Release’ when at least two of the three fields were reported based on ‘Catch’ being equal to the sum of ‘Harvest’ and ‘Release’. Rate based calculations included various fish and preference metrics divided by effort metrics (hours and outings), survey area, or survey duration. We calculated percentage fields by dividing a record’s value with the sum of all values for the field by survey and multiplying by 100 (i.e., percentage of harvest is the harvest value for a taxon divided by the sum of all taxa harvested within a survey multiplied by 100). The metadata for all fields which contain a calculation include a description of what fields the calculation was based on and what operations were applied in the field description which allow for replication of methodology. We summarized metrics associated with taxa in the fish and angler preference tables to higher taxonomic levels by summing estimates for all reported taxa within a classification (i.e., harvest for Centrarchidae calculated by summing reported harvest for all estimates of taxa within the Centrarchidae family). All taxonomic relationships are documented within the ‘Taxa_Data’ table to allow for replication and validation of methodology^30^.

## Data Records

CreelCat is stored in the U.S. Geological Survey (USGS) ScienceBase data repository (10.5066/P9DSOPHD)^30^, and is also accessible via the CreelCat web application (https://rconnect.usgs.gov/CreelCat). As of May 1, 2023, CreelCat (Version 1.0) contains 14,729 inland creel and angler survey records compiled from 35 natural resource management agencies across the United States.

### Data structure

We structured the database as a set of eight ‘.csv’ files containing tabular data as well as three shapefiles, which can be downloaded individually or via a single geopackage file, as well as an ‘.xml’ file containing FGDC compliant metadata for all tabular and geospatial data or queried and downloaded via the CreelCat web application. Tabular data include information about survey characteristics (Survey_Data.csv; 14,729 records), angler effort (AngEffort_Data.csv; 13,576 records), catch and harvest (FishDataCompiled.csv; 235,015 records), angler demographics (Demographic_Data.csv; 409 records), angler preferences (AngPrefDataCompiled.csv; 27,250 records), taxonomic classifications (Taxa_Data.csv; 20 subspecies and variants, 149 species, 66 genera, 25 families, and 5 non-taxonomic groups), issues with catch and harvest (Fish_Attribution_Issues.csv; 104,142), and issues with angler preference (AngPref_Attribution_Issues.csv; 18,946). Tables may be linked using the unique identifier found in the ‘Survey_ID’ field present in each table (except the ‘Taxa_Data.csv’ table which contains taxonomic relationships which are not specific to any given survey). To link fish or angler preference data with their respective attribution issue tables, both ‘Survey_ID’ and ‘Taxa’ or ‘Target_Taxa’ should be used in the join. Geospatial representation of survey locations are expressed as a polygons shapefile (n = 2159) for surveys of lakes, reservoirs, ponds, and tailraces, a lines shapefile (n = 161) for rivers and streams, and a points shapefile (n = 2755) for the locations of all georeferenced surveys. In most cases, we georeferenced the location and extent of surveys (n = 2320 out of 3034) but in some cases surveys were unable to be spatially attributed. Geospatial data can be joined to the tabular data using the ‘WB_ID’ field present in all shapefiles as well as the ‘Survey_Data.csv’ table. FGDC compliant metadata is contained in the ‘CreelCat_Metadata.xml’ file.

### Tabular fields

As of May 1, 2023, CreelCat contains a total of 235 fields associated with survey and waterbody characteristics (57), angler effort (17), catch and harvest of fish (55), angler demographics (7), angler preferences (52), catch and harvest attribution issues (16), angler preference attribution issues (13), and taxonomic relationships (18; Supplementary Table 1).

### Geospatial files

As of May 1, 2023, CreelCat contains geospatial information for 13,213 (out of 14,729) surveys. All geospatial information is represented using shapefiles. Approximate spatial extents of 7,502 surveys of lakes, reservoirs, ponds, and tailraces are represented as polygons. Lines are used to represent the approximate spatial extent of 244 surveys of rivers and streams. Point locations are representative of all surveys represented as lines and polygons, as well as surveys for which the spatial extent of a survey is not determined.

## Technical Validation

Validation of the data included in CreelCat occurred through quality assurance procedures utilized by the agencies providing the data, during an initial review of received datasets, during data entry, through a review of the completed dataset, with additional validation performed for geospatial attribution of survey data, and an external review of the complete dataset.

### Agency procedures

While methods for internal data validation vary across agencies, units, and time, care was taken to ensure only records which met the quality standards of the providing agency were met. This involved receiving written approval to publicly release the data we received and reviewing any provided information which indicated the status of any internal data reviews. Data provided to us which had not been vetted and approved for release by the providing agency (i.e., survey records flagged as preliminary, unapproved, in-review, etc.) were excluded from CreelCat.

### Initial assessment

We processed all data received for CreelCat using assessment procedures to ensure the data were reliable, complete, and interpretable. We have written permission to make data publicly available for all data included in the database. We assessed data reliability by reviewing the dataset for any correspondence, comments, or text which indicated uncertainty around the validity of the data. Datasets which were determined to be unreliable were not included in CreelCat based on correspondence with data providers or comments in source data. We assessed data completeness by reviewing the dataset and any associated metadata to determine whether information was available that documented the type of survey and associated methodology, when the survey was conducted and for what time period the estimates were attributable, and whether geospatial data were available to assign the spatial extent of surveys. When this information was not available, we contacted data providers to request the additional information. If additional data were not available, those datasets were then excluded from CreelCat. We resolved data interpretability issues via an initial assessment which required units for all numeric fields to be determined and documented, ambiguous field names to be resolved (i.e., was a field called “Harvest Rate” the number of fish harvested per hour of fishing effort, the rate of harvest for captured fish, or something else?), and documentation for any non-standard naming systems to be provided (i.e., taxa codes).

### Data entry validation

We conducted data validation during data entry to ensure accurate transcription of the data and ensure values fell within reasonable bounds. We reviewed records upon entry to confirm all values were placed in appropriate columns. We evaluated any entries that contained values which were substantially higher or lower for accuracy first by the person entering the data, then by the database manager to ensure accuracy. In some cases, potential issues with the source data were identified and noted using contextual or warning fields rather than discarding the data. This included noting surveys where the timing, location, or spatial extent of the survey was either ‘uncertain’ (we lacked confidence in the accuracy of reported information), or ‘unknown’ (reported information was inadequate or missing so no value was recorded in CreelCat). Another issue was that although estimates of catch should be equal to the sum of harvest and release, at times they were not. In cases where there was an imbalance among reported estimates of catch, harvest, and release, we identified the values as potentially erroneous and calculated the absolute and proportional difference between catch and harvest and release. In some cases of imbalance, the issue may stem from minor discrepancies, such as rounding, which does not necessarily invalidate the estimates, while in other cases large differences may exist due to computational or transcription errors in the source dataset. Users of the dataset should consider whether, and to what degree, an imbalance among catch, harvest, and release metrics is acceptable and retain only those data meeting that standard within their analysis.

We performed taxonomic validation for all entries to ensure that names were consistent across all surveys contained in CreelCat and validated names with the ITIS database^31^. Surveys attribute both catch/harvest and angler preference across a variety of taxonomic levels and because of this care needs to be taken to ensure the data contained in CreelCat is properly interpreted. Metrics can be pooled to higher taxonomic levels but cannot be attributed to lower taxonomic levels. In some cases, metrics are reported at higher taxonomic levels either due to the limitation of anglers to make species level identifications or the decision by agencies to manage at higher taxonomic levels^34–36^. For example, catch estimates for a genus such as ‘*Micropterus* (Black Bass)’ can be pooled with all other reported taxa within the Centrarchid family to get a family level estimate of ‘Catch’, however, the value reported at the genus level cannot be attributed to calculate species level estimates such as ‘*Micropterus salmoides* (Largemouth Bass)’. Any instances where a reported value has potential lower-level attributions that cannot be determined are flagged as unattributed taxa in the taxa issues table. For example, all *Micropterus* species (i.e., *M. salmoides*, *M. dolomieu*, *M. punctulatus*) are flagged as ‘Unattributed’ when a survey reports metrics more generally for the *Micropterus* genus. In some cases, metrics are reported at multiple, overlapping taxa in a single survey. For example, some anglers may be able to accurately report that they captured a certain number of *Pomoxis annularis* (White Crappie) and a certain number of *P. nigromaculatus* (Black Crappie), another angler may only be able to report the total number of *Pomoxis* spp. (Crappie) which they caught. In these cases, the values reported at the species level are identified as being partially attributed because although some attribution occurred by anglers who reported taxa at the more specific taxonomic level, other reports were at the more generic level and therefore additional catch of those species may have occurred but could not be fully attributed. Finally, there are also cases where records are flagged as containing missing attributions, which occurs when metrics are populated for some taxa within a group, but others have no value (NA) reported which means the value cannot be summed to generate an estimate. For example, a survey may have catch estimates for *Ictalurus furcatus* (Blue catfish), *I. punctatus* (Channel Catfish), and *Pylodictis olivaris* (Flathead Catfish) but only has harvest estimates available for Blue and Flathead Catfish and an NA reported for Channel Catfish. Because of this a summed estimate of *Ictaluridae* (Catfish) harvest cannot be computed, despite the knowledge that harvest occurred for Blue and Flathead Catfish because harvest was not reported for Channel Catfish.

### Complete dataset review

After we completed data entry, we reviewed the data to ensure both survey and waterbody identifiers were truly unique. We also reviewed the dataset to ensure that duplicate entries did not occur within any tables. We ensured that multiple rows were not present for each survey identifier in the survey data, effort data, and demographic data tables, and no duplication of any combination of survey identifier and taxa in the fish and angler preference data tables. All fields containing factors and names were reviewed to ensure values matched those found in the associated list of potential values. When values were not found on the list, we corrected values which were incorrect (e.g., misspelling, incorrect naming convention) and added values where appropriate (i.e., new taxa). Next, we evaluated all numeric values to determine whether they fell within expected bounds. This included identifying any cases of negative values for fields in which only positive values should exist and percentage values over 100. In some cases, we determined values outside of the expected bounds to be legitimate (i.e., percentages slightly greater than 100 due to rounding of component values) and retained them; however, we removed records with values outside of expected bounds which could not be validated or corrected. We then sorted each field within the dataset to identify both high and low outlier values. We validated the highest and lowest sets of values for each field by reviewing the source data to ensure validity, and corrected when possible or removed any records containing errors.

### Geospatial validation

We assessed the validity of geospatial representation of survey locations and extents using different methods depending on the approach used to attribute spatial data. All spatial data were transformed to CRS: 4326 to ensure matching, appropriate projections were used^37^. We evaluated all locations to determine whether they fell within the political boundaries of the data provider. We validated locations of any waterbodies outside of the political boundaries of the data provider against descriptions in the source data. In some cases, border waterbodies fall partially outside provider boundaries but are still legitimate spatial attributions. In cases where geospatial data for waterbodies came in the form of a publicly available geospatial dataset, no additional validation was performed. When we manually attributed the spatial representation of waterbodies, we assigned correct locations by evaluating contextual information such as waterbody surface area as well as descriptive information such as nearby place names (e.g., roads, cities, parks). When waterbody locations were provided by spatial coordinates, we validated by determining whether the waterbody name at the provided coordinates matched the waterbody name reported in the survey data.

### External review

As a U.S. Geological Survey data release this dataset was also subjected to an external review to ensure that the dataset met all criteria for quality and reliability outlined by U.S. Geological Survey fundamental science practices. This review included a complete review of the tabular, geospatial and metadata by an expert who was not affiliated with the project. This expert reviewed the metadata for completeness, accuracy, interpretability, and compliance with Federal Geographic Data Committee (FGDC) standards. In their review of the data records they verified data types were consistent and correct, numeric values were within reasonable ranges, factors were consistently classified, and that the structure of the data was appropriate and consistent. The review of the geospatial data included validation of positional accuracy, consistency and appropriateness of projections, and relationship with the associated tabular data. Once revisions based on this review were completed the data release was then reviewed for approval by agency administrators.

## Usage Notes

The angler survey data compiled for CreelCat is intended to support large-scale research and management investigations of inland recreational fisheries in the United States. A static version of CreelCat (Version 1.0), referenced in this manuscript, is stored in the USGS ScienceBase data repository (10.5066/P9DSOPHD)^30^. The dataset will be preserved as published, however updated versions of the dataset will be posted to ScienceBase with incremented versioning numbers and a notice and link will be posted under the Version 1.0 landing page to inform users that a newer version of the dataset exists if they wish to use it. The dataset can be used in a variety of ways ranging from large-scale evaluations of national recreational fisheries issues to assessing conditions in a single waterbody at a specific point in time. Because of the variability in survey methodology including timing and duration of surveys, differences in data collection and analysis, terminology and classification methods, and several other factors, we recommend that care should be given to ensure data are used appropriately in all analyses. We recommend viewing the dataset within the associated application (https://rconnect.usgs.gov/CreelCat/) to view customized notifications related to discrepancies in survey characteristics, taxonomic attribution issues, and other important data considerations. We recommend all users of CreelCat review the ‘Data Considerations’ section of the user guide for detailed descriptions of a variety of important considerations for working with this dataset.

### Supplementary information

Supplementary Materials

## Data Availability

No custom code is being published related to the creation of this dataset, however any fields that involve calculations have been documented in the metadata to allow users of the dataset to replicate and evaluate the calculations which were made to produce the dataset.
