# Supplementary material for: CreelCat, a Catalog of United States Inland Creel and Angler Survey Data
Source: Sci Data. 2023 Nov 3;10:762. doi: 10.1038/s41597-023-02523-2 (PMC10624813; doi:10.1038/s41597-023-02523-2)
Supplement: Supplementary file 1 — Supplementary Materials [file 41597_2023_2523_MOESM1_ESM.docx]

*Supplementary Tables*

Supplementary Table 1: Names and descriptions of all fields in the United States Inland Creel and Angler Survey Catalog (CreelCat version 1.0: https://doi.org/10.5066/P9DSOPHD) organized by table.

| Field_Name | Field_Description |
| --- | --- |
| **Survey Information** |  |
| Survey_ID | Unique identifier created to link surveys across database tables (Formatted as: [WB_ID]_[Year]_*Unique Letter*) |
| Source_Survey_ID | Survey identifier associated with the survey from provider dataset |
| State | Name of state (or territory, or Washington DC) associated with the survey data |
| State_Ab | US Postal Service abbreviation for the state associated with the survey |
| Year | Year in which the survey began |
| Start_Date | Date survey period began (Formatted as Month/Day/Year) |
| End_Date | Date survey period ended (Formatted as Month/Day/Year) |
| Season | Classification used for timing of the survey period |
| Reported_Duration | Reported number of days in the survey period |
| Timing_Warn | Identifies surveys which have "Unknown" or "Uncertain" timing characteristics |
| Timing_Comment | Descriptive comments related to the timing of the survey |
| Waterbody_Name | Name of surveyed waterbody as provided by the data source |
| WB_ID | A unique code used to identify a waterbody (Formatted as [State_Ab]_*4 digit number*) |
| Source_Waterbody_ID | Identifier attributed to waterbodies by the data provider (typically state-specific waterbody codes) |
| Reported_Acres | Surface area of waterbody within the bounds of the survey as reported in the source data |
| Reported_Miles | Length of stream within survey bounds as reported in the source data |
| Survey_Type | Identifies the approach used to conduct the survey (ie. Angler Intercept, Mail Survey, etc.) |
| Interview_Method | Classification of the approach used for interviewing anglers |
| Count_Method | Classification of the approach used for counting anglers |
| Estimate_Method | Description of approach used for generating estimates |
| Protocol_Comments | Descriptive comments related to the timing of the survey |
| Survey_Gaps | Description of periods within the overall survey period which not included in generating the estimate (ie. an entry of "July" indicates that the month of July was not included in the estimate) |
| Lost_Duration | The number of days which were not included as part of the survey period when generating estimates. This value was subtracted from the calculated duration to yield the actual number of days (Duration) represented by the survey. |
| Location_Warn | Identifies surveys for which the location of the survey is either "Unknown" (No survey location identified) or "Uncertain" (Uncertainty exists as to whether the survey location provided is correct). |
| Extent_Warn | Surveys flagged with 'Uncertain' have had their spatial extent attributed to a waterbody but there is uncertainty if it is accurate, surveys flagged with 'Unknown' have not been assigned an survey extent |
| Survey_Extent_Class | Classifications of survey extent. Can be 'Single' (complete survey of single waterbody), 'Multiple' (complete survey of multiple waterbodies), 'Partial' (only a portion of single (or multiple waterbodies) included in survey such as a portion of a reservoir, or a stretich of stream, or 'Multiclass' (survey that contains both lentic and lotic waterbodies). |
| Focal_Species | Identifies whether survey captures information on all captured species or focus only on a subset of taxa |
| Survey_Citation | Citation describing the methodology used in the survey |
| Report_Citation | Citation for the report the data came from |
| Agency | Name of agency which provided the data |
| Data_Source_Unit | Branch, Division, Unit, Bureau, etc. of agency which provided the data |
| Survey_Link | Link providing access to source copies of surveys |
| Survey_Comments | Descriptive comments related to the survey data |
| Survey_Extent_Comment | Descriptive comments related to the spatial extent of a survey. |
| WB_Type | Classified as either "Lakes, Reservoirs, Ponds, and Tailraces" or "Rivers and Streams" |
| County | Name of county with the largest spatial overlap with the surveyed waterbody/waterbodies |
| GNIS_Name | Name attributed to waterbodies within the USGS Geographic Names Information System |
| GNIS_ID | Unique code attributed to waterbodies within the USGS Geographic Names Information System |
| Perm_ID | Unique code used to identify waterbodies in the National Hydrography Dataset ("Permanent Identifier" field) |
| Calc_Acres | Survey extent in acres of surveyed waterbody surface area calculated from the associated geospatial data |
| Calc_Miles | Survey extent in linear stream length measured in miles from the associated geospatial data |
| Up_Lat | Approximate latitude of the upstream most extent of the survey |
| Up_Lon | Approximate longitude of the upstream most extent of the survey |
| Down_Lat | Approximate latitude of the downstream most extent of the survey |
| Down_Lon | Approximate longitude of the downstream most extent of the survey |
| Lat | Latitude of surveyed waterbody |
| Lon | Longitude of surveyed waterbody |
| NHD_Acres | Area measured in acres of the NHD waterbody with the greatest overlap with the survey extent |
| Area_Diff | Difference in acres between the NHD derived and CreelCat geospatial areas; [NHD_Acres]-[Calc_Acres] |
| Area_Diff_Percent | Percent difference between the calculated area and NHD area; [Calc_Acres]/[NHD_Acres]*100 |
| Survey_Acres | Acreage assigned to survey extent. When survey area was reported in the source data [Reported_Acres] that value was used for this field, otherwise calculated area from the geospatial attribution was used [Calc_Acres] |
| Survey_Miles | Miles assigned to survey extent. When survey length was reported in the source data [Reported_Miles] that value was used for this field, otherwise calculated area from the geospatial attribution was used [Calc_Miles] |
| Start_Month | Month survey began |
| End_Month | Month survey ended |
| Duration | Length of the associated survey period given in days |
| County_ID | Unique identifier for counties formatted as [State_Ab]_[County] |
| Waterbody_Unique_Name | Unique name for distinct waterbodies formatted as [Waterbody_Name]_[State_Ab]_[County]_*Unique Character* |
|  |  |
| **Effort Info** |  |
| Survey_ID | Unique identifier created to link surveys across database tables (Formatted as: [WB_ID]_[Year]_*Unique Letter*) |
| Effort_Hours | Estimate of the number of cumulative hours spent angling by all anglers during the survey period |
| Effort_Hours_SE | Standard error for the estimate of cumulative effort hours |
| Effort_Outings | Estimate of the cumulative number of outings by anglers during the survey period (Use [Effort_Outings_Type] to determine whether unit is 'Days' or 'Trips') |
| Effort_Outings_Type | Classifies the type of outings being reported in the 'Effort_Outings' field ('Days' are the number days spent fishing by anglers; 'Trips' the number angling trips taken by anglers, can be multiple in a day) |
| Effort_Outings_SE | Standard error for the estimate of the cumulative number of outings |
| Effort_Anglers | Estimate of the number anglers for the entire survey period |
| Effort_Hours_Raw | Number of hours spent angling by interviewed anglers (Not expanded to non-interviewees) |
| Effort_Outings_Raw | Count of number of outings based on interviews (Not expanded to non-interviewees) |
| Effort_Anglers_Raw | Count of number of anglers interviewed (Not expanded to non-interviewees) |
| Effort_Comments | Descriptive comments related to the angler effort data |
| Effort_Hours_Per_Day | Estimate of effort hours per day; calculated as [Effort_Hours]/[Duration] |
| Effort_Hours_Per_Acre | Estimate of effort hours per acre; calculated as [Effort_Hours]/[Survey_Acres] |
| Effort_Hours_Per_Day_Per_Acre | Estimate of effort hours per day per acre; calculated as [Effort_Hours]/[Duration]/[Survey_Acres] |
| Effort_Outings_Per_Day | Estimate of outings per day; calculated as [Effort_Outings]/[Duration] |
| Effort_Outings_Per_Acre | Estimate of outings per acre; calculated as [Effort_Outings]/[Survey_Acres] |
| Effort_Outings_Per_Day_Per_Acre | Estimate of outings per day per acre; calculated as [Outings]/[Duration]/[Survey_Acres] |
|  |  |
| **Angler Preference** |  |
| Survey_ID | Unique identifier created to link surveys across database tables (Formatted as: [WB_ID]_[Year]_*Unique Letter*) |
| Target_Taxa | Scientific nomenclature followed by common name for monophyletic taxa, for non-monphyletic groups the common name for the group is used (ie. 'Panfish') |
| Target_Taxa_Class | Taxonomic level of target taxa (Subspecies/Variant, Species, Genus, Family) or Group for non-monophyletic taxa groupings |
| Target_TSN | Taxonomic serial number associated with the taxa derived from the Integrated Taxonomic Information System (ITIS) |
| Target_Taxa_Source | Identifes whether the metrics for a record are those which were reported in the source data or attributed to a different taxonomic level via calculation (ie. Value for 'Lepomis' (genus) calculated by summing reported values for all species within the genus) |
| Target_Percent | Reported or calculated percentage of effort spent targeting a given taxa |
| Target_Percent_Calc | Description of the method which was used for calculating target percentage for a given record |
| Target_Hours | Estimated cumulative number of hours spent by anglers targeting a given taxa |
| Target_Hours_SE | Standard error associated with the estimate for the cumulative number of hours spent targeting the associated taxa |
| Target_Outings | Estimated number of outings ('Days' or 'Trips') spent by anglers targeting a given taxa |
| Target_Outings_Type | Classifies the type of outings being reported in the 'Target_Outings' field ('Days' are the cumulative number days spent by anglers targeting the taxa; 'Trips' the cumulative number trips taken by anglers targeting the taxa, can be multiple in a day) |
| Target_Outings_SE | Standard error associated with the estimate for number of hours spent targeting the associated taxa |
| Target_Percent_Raw | Reported values of the percent of effort spent targeting the associated taxa |
| Target_Catch | Estimate of total catch by anglers targeting the associated taxa |
| Target_Harvest | Estimate of total harvest by anglers targeting the associated taxa |
| Target_Release | Estimate of total release by anglers targeting the associated taxa |
| Target_Harvest_SE | Standard error of the estimate of targeted harvest |
| Target_Release_SE | Standard error of the estimate of targeted release |
| Target_Catch_SE | Standard error of the estimate of targeted catch |
| Ang_Pref_Comments | Descriptive comments related to the angler preference data |
| Unbalanced_Target_Estimates_Value | The difference between estimated targeted catch and the sum of targeted harvest and release estimates; calculated as [Target_Catch]-([Target_Harvest]+[Target_Release]) |
| Unbalanced_Target_Estimates_Percent | The percentage difference between estimated targeted catch and the sum of targeted harvest and release estimates; calculated as [Target_Catch]/([Target_Harvest]+[Target_Release])*100-100 |
| Reported_Target_Estimate_Warning | Identifier of records in which the the 'Unbalance_Target_Estimate_Value' field is not equal to 0 |
| Target_Hours_Per_Day | Estimate of target hours per day; calculated as [Target_Hours]/[Duration] |
| Target_Hours_Per_Acre | Estimate of target hours per acre; calculated as [Target_Hours]/[Survey_Acres] |
| Target_Hours_Per_Day_Per_Acre | Estimate of target hours per day per acre; calculated as [Target_Hours]/[Duration]/[Survey_Acres] |
| Target_Outings_Per_Day | Estimate of target outings per day; calculated as [Target_Outings]/[Duration] |
| Target_Outings_Per_Acre | Estimate of target outings per acre; calculated as [Target_Outings]/[Survey_Acres] |
| Target_Outings_Per_Day_Per_Acre | Estimate of target outings per day per acre; calculated as [Target_Outings]/[Duration]/[Survey_Acres] |
| Target_Released_Percent | Percentage of targeted captured individuals which were released; calculated as [Target_Release]/[Target_Catch]*100 |
| Target_Harvest_Percent | Percentage of targeted captured individuals which were harvested; calculated as [Target_Release]/[Target_Catch]*100 |
| Target_Catch_Per_Day | Estimate of target catch per day; calculated as [Target_Catch]/[Duration] |
| Target_Harvest_Per_Day | Estimate of target harvest per day; calculated as [Target_Harvest]/[Duration] |
| Target_Release_Per_Day | Estimate of target release per day; calculated as [Target_Release]/[Duration] |
| Target_Release_Per_Hour | Estimate of target release per target hour; calculated as [Target_Release]/[Target_Hours] |
| Target_Catch_Per_Hour | Estimate of target catch per target hour; calculated as [Target_Catch]/[Target_Hours] |
| Target_Harvest_Per_Hour | Estimate of target harvest per target hour; calculated as [Target_Harvest]/[Target_Hours] |
| Target_Release_Per_Outing | Estimate of target release per target outing; calculated as [Target_Release]/[Target_Outings] |
| Target_Catch_Per_Outing | Estimate of target catch per target outings; calculated as [Target_Catch]/[Target_Outings] |
| Target_Harvest_Per_Outing | Estimate of target harvest per target outing; calculated as [Target_Harvest]/[Target_Outings] |
| Target_Catch_Per_Acre | Estimate of target catch per survey acre; calculated as [Target_Catch]/[Survey_Acres] |
| Target_Harvest_Per_Acre | Estimate of target harvest per survey acre; calculated as [Target_Harvest]/[Survey_Acres] |
| Target_Release_Per_Acre | Estimate of target release per survey acre; calculated as [Target_Release]/[Survey_Acres] |
| Target_Catch_Per_Hour_Per_Acre | Estimate of target catch per target hour per survey acre; calculated as [Target_Catch]/[Target_Hours]/[Survey_Acres] |
| Target_Release_Per_Hour_Per_Acre | Estimate of target release per target hour per survey acre; calculated as [Target_Release]/[Target_Hours]/[Survey_Acres] |
| Target_Harvest_Per_Hour_Per_Acre | Estimate of target harvest per target hour per survey acre; calculated as [Target_Harvest]/[Target_Hours]/[Survey_Acres] |
| Target_Catch_Per_Outing_Per_Acre | Estimate of target catch per target outing per survey acre; calculated as [Target_Catch]/[Target_Outings]/[Survey_Acres] |
| Target_Release_Per_Outing_Per_Acre | Estimate of target release per target outings per survey acre; calculated as [Target_Release]/[Target_Outings]/[Survey_Acres] |
| Target_Harvest_Per_Outing_Per_Acre | Estimate of target harvest per target outings per survey acre; calculated as [Target_Harvest]/[Target_Outings]/[Survey_Acres] |
| Target_Catch_Per_Day_Per_Acre | Estimate of target catch per day per survey acre; calculated as [Target_Catch]/[Duration]/[Survey_Acres] |
| Target_Release_Per_Day_Per_Acre | Estimate of target release per day per survey acre; calculated as [Target_Release]/[Duration]/[Survey_Acres] |
| Target_Harvest_Per_Day_Per_Acre | Estimate of target harvest per day per survey acre; calculated as [Target_Harvest]/[Duration]/[Survey_Acres] |
|  |  |
| **Angler Demographics** |  |
| Survey_ID | Unique identifier created to link surveys across database tables (Formatted as: [WB_ID]_[Year]_*Unique Letter*) |
| Mean_Age | The average age of anglers |
| Per_Female | Percentage of anglers classified as female |
| Per_Male | Percentage of anglers classified as male |
| Per_Resident | Percentage of anglers classified as resident |
| Per_Nonresident | Percentage of anglers classified as non-resident |
| Demographic_Comments | Descriptive comments related to the demographic data |
|  |  |
| **Fish Metrics** |  |
| Survey_ID | Unique identifier created to link surveys across database tables (Formatted as: [WB_ID]_[Year]_*Unique Letter*) |
| Taxa | Scientific nomenclature followed by common name for monophyletic taxa, for non monphyletic taxa the common name for the group (ie. panfish) |
| Taxa_Class | Taxonomic level of target taxa (Subspecies/Variant, Species, Genus, Family) or Group for non-monophyletic taxa groupings |
| TSN | Taxonomic serial number associated with the taxa derived from the Integrated Taxonomic Information System (ITIS) |
| Taxa_Source | Identifies whether the metrics for a record are those which were reported in the source data or attributed to a different taxonomic level via calculation (ie. Value for 'Lepomis' (genus) calculated by summing reported values for all species within the genus) |
| Catch | Total catch estimate, represented as number of individuals, for the associated taxa |
| Harvest | Total harvest estimate, represented as number of individuals, for the associated taxa |
| Release | Total release estimate, represented as number of individuals, for the associated taxa |
| Catch_SE | Standard error of the catch estimate |
| Harvest_SE | Standard error of the harvest estimate |
| Release_SE | Standard error of the release estimate |
| Unbalanced_Estimates_Value | The difference between estimated catch and the sum of harvest and release estimates; calculated as [Catch]-([Harvest]+[Release]) |
| Unbalanced_Estimates_Percent | The percentage difference between estimated catch and the sum of harvest and release estimates; calculated as [Catch]/([Harvest]+[Release])*100-100 |
| Reported_Estimate_Warning | Identifier of records in which the the 'Unbalance_Estimate_Value' field is not equal to 0 |
| Released_Percent | The percentage of captured individuals which were released; calculated as [Release]/[Catch]*100 |
| Harvested_Percent | The percentage of captured individuals which were harvested; calculated as [Harvest]/[Catch]*100 |
| Catch_Per_Day | Estimate of catch per day; calculated as [Catch]/[Duration] |
| Harvest_Per_Day | Estimate of harvest per day; calculated as [Harvest]/[Duration] |
| Release_Per_Day | Estimate of release per day; calculated as [Release]/[Duration] |
| Release_Per_Hour | Estimate of release per hour; calculated as [Release]/[Effort_Hours] |
| Catch_Per_Hour | Estimate of catch per hour; calculated as [Catch]/[Effort_Hours] |
| Harvest_Per_Hour | Estimate of harvest per hour; calculated as [Harvest]/[Effort_Hours] |
| Release_Per_Outing | Estimate of release per outing; calculated as [Release]/[Effort_Outings] |
| Catch_Per_Outing | Estimate of release per outing; calculated as [Catch]/[Effort_Outings] |
| Harvest_Per_Outing | Estimate of harvest per outing; calculated as [Harvest]/[Effort_Outings] |
| Catch_Per_Acre | Estimate of catch per acre; calculated as [Catch]/[Survey_Acres] |
| Harvest_Per_Acre | Estimate of harvest per acre; calculated as [Harvest]/[Survey_Acres] |
| Release_Per_Acre | Estimate of release per acre; calculated as [Release]/[Survey_Acres] |
| Catch_Per_Hour_Per_Acre | Estimate of catch per hour per acre; calculated as [Catch]/[Effort_Hours]/[Survey_Acres] |
| Release_Per_Hour_Per_Acre | Estimate of release per hour per acre; calculated as [Release]/[Effort_Hours]/[Survey_Acres] |
| Harvest_Per_Hour_Per_Acre | Estimate of harvest per hour per acre; calculated as [Harvest]/[Effort_Hours]/[Survey_Acres] |
| Catch_Per_Outing_Per_Acre | Estimate of catch per outing per acre; calculated as [Catch]/[Effort_Outings]/[Survey_Acres] |
| Release_Per_Outing_Per_Acre | Estimate of release per outing per acre; calculated as [Release]/[Effort_Outings]/[Survey_Acres] |
| Harvest_Per_Outing_Per_Acre | Estimate of harvest per outing per acre; calculated as [Harvest]/[Effort_Outings]/[Survey_Acres] |
| Catch_Per_Day_Per_Acre | Estimate of catch per day per acre; calculated as [Catch]/[Duration]/[Survey_Acres] |
| Release_Per_Day_Per_Acre | Estimate of release per day per acre; calculated as [Release]/[Duration]/[Survey_Acres] |
| Harvest_Per_Day_Per_Acre | Estimate of harvest per day per acre; calculated as [Harvest]/[Duration]/[Survey_Acres] |
| Harvested_Weight | Estimate of the total harvested weight, in pounds, for the associated taxa; calculated as [Harvest]*[Mean_Weight_Harvest] |
| Harvested_Weight_Per_Day | Estimate of the total harvested weight, in pounds, for the associated taxa per day; calculated as [Harvest]*[Mean_Weight_Harvest]/[Duration] |
| Harvested_Weight_Per_Hour | Estimate of the total harvested weight, in pounds, for the associated taxa per hour; calculated as [Harvest]*[Mean_Weight_Harvest]/[Effort_Hour] |
| Harvested_Weight_Per_Outing | Estimate of the total harvested weight, in pounds, for the associated taxa per outing; calculated as [Harvest]*[Mean_Weight_Harvest]/[Effort_Outing] |
| Harvested_Weight_Per_Acre | Estimate of the total harvested weight, in pounds, for the associated taxa per acre; calculated as [Harvest]*[Mean_Weight_Harvest]/[Survey_Acres] |
| Percent_of_Catch | The percentage of total catch (for all taxa) attributable to the associated taxa for a given survey; calculated as [Catch (Associated Taxa)]/[Catch (Sum All Taxa)] |
| Percent_of_Harvest | The percentage of total harvest (for all taxa) attributable to the associated taxa for a given survey; calculated as [Harvest (Associated Taxa)]/[Harvest (Sum All Taxa)] |
| Percent_of_Release | The percentage of total release (for all taxa) attributable to the associated taxa for a given survey; calculated as [Release (Associated Taxa)]/[Release (Sum All Taxa)] |
| Mean_Length_Catch | Mean length, in inches, of captured fish for the associated taxa |
| Mean_Length_Harvest | Mean length, in inches, of captured fish for the associated taxa |
| Mean_Length_Release | Mean length, in inches, of captured fish for the associated taxa |
| Mean_Weight_Catch | Mean weight, in pounds, of captured fish for the associated taxa |
| Mean_Weight_Harvest | Mean weight, in pounds, of harvested fish for the associated taxa |
| Mean_Weight_Release | Mean weight, in pounds, of released fish for the associated taxa |
| Catch_Raw | Number of individuals, of the associated taxa, captured by interviewed anglers (Not expanded to non-interviewees) |
| Harvest_Raw | Number of individuals, of the associated taxa, harvested by interviewed anglers (Not expanded to non-interviewees) |
| Release_Raw | Number of individuals, of the associated taxa, released by interviewed anglers (Not expanded to non-interviewees) |
| Fish_Comments | Descriptive comments related to the fish data |
|  |  |
| **Taxa Data** |  |
| Subspecies_And_Variants_TSN | Taxonomic serial number from the U.S. Geological Survey Integrated Taxonomic Information System for the associated subspecies or variant |
| Species_TSN | Taxonomic serial number from the U.S. Geological Survey Integrated Taxonomic Information System for the associated species |
| Genus_TSN | Taxonomic serial number from the U.S. Geological Survey Integrated Taxonomic Information System for the associated genus |
| Family_TSN | Taxonomic serial number from the U.S. Geological Survey Integrated Taxonomic Information System for the associated family |
| Subspecies_And_Variants_Choice | Combined taxonomic and common name for the associated subspecies or variant |
| Species_Choice | Combined taxonomic and common name for the associated species |
| Genus_Choice | Combined taxonomic and common name for the associated genus |
| Family_Choice | Combined taxonomic and common name for the associated family |
| Species_Sci | Scientific name of the associated species |
| Species_Com | Common name of the associated species |
| Family_Sci | Scientific name of the associated family |
| Family_Com | Common name of the associated family |
| Genus_Sci | Scientific name of the associated genus |
| Genus_Com | Common name of the associated genus |
| Species | Species scientific name |
| Subspecies_Variant_Sci | Scientific name of the associated subspecies or variant |
| Subspecies_Variant_Com | Common name of the associated subspecies or variant |
| Hybrid | Identifier of hybrid taxa |
|  |  |
| **Fish Attribution Issues** |  |
| Survey_ID | Unique identifier created to link surveys across database tables (Formatted as: [WB_ID]_[Year]_*Unique Letter*) |
| Taxa | Name of taxa associated with the record |
| TSN | Taxonomic serial number for the taxa associated with the record |
| Unattributed_Taxa | Identifier or records containing unattributed taxa |
| Attributed_Taxa | Name of attributed taxa associated with unattributed taxa |
| Attributed_TSN | Taxonomic serial number of the attributed taxa associated with the unattributed taxa |
| Partially_Attributed_Taxa | Identifier or records containing partially attributed taxa |
| Missing_Harvest | Identifies records with missing harvest values due to presence of NA for at least one lower level taxa |
| Missing_Catch | Identifies records with missing catch values due to presence of NA for at least one lower level taxa |
| Missing_Release | Identifies records with missing release values due to presence of NA for at least one lower level taxa |
| Unbalanced_Estimates | Identifies records in which the sum of estimates for harvest and release are not equal to the estimate for catch |
| Unbalanced_Estimates_Value | Difference between the catch and sum of harvest and release estimates; [Catch]-([Harvest]+[Release]) |
| Unbalanced_Estimates_Percent | Percent difference between catch and sum of harvest and release estimates; [Catch]/([Harvest]+[Release])*100 |
| Catch | Catch estimate for associated taxa |
| Harvest | Harvest estimate for associated taxa |
| Release | Release estimate for associated taxa |
|  |  |
| **Angler Preference Attribution Issues** |  |
| Survey_ID | Unique identifier created to link surveys across database tables (Formatted as: [WB_ID]_[Year]_*Unique Letter*) |
| Target_Taxa | Name of target taxa associated with the record |
| Target_TSN | Taxonomic serial number for the target taxa associated with the record |
| Unattributed_Target_Taxa | Identifier or records containing unattributed target taxa |
| Attributed_Target_Taxa | Name of attributed taxa associated with unattributed target taxa |
| Attributed_Target_TSN | Taxonomic serial number of the attributed target taxa associated with the unattributed target taxa |
| Partially_Attributed_Target_Taxa | Identifier or records containing partially attributed target taxa |
| Missing_Target_Hours | Identifies records with missing effort hours estimate due to presence of NA for at least one lower level taxa |
| Missing_Target_Outings | Identifies records with missing effort outings estimate due to presence of NA for at least one lower level taxa |
| Missing_Target_Harvest | Identifies records with missing targeted harvest estimate due to presence of NA for at least one lower level taxa |
| Missing_Target_Catch | Identifies records with missing targeted catch estimate due to presence of NA for at least one lower level taxa |
| Missing_Target_Release | Identifies records with missing targeted release estimate due to presence of NA for at least one lower level taxa |
| Missing_Target_Percent | Identifies records with missing target percent value due to presence of NA for at least one lower level taxa |
